# CT and Fluorine-18-Fluorodeoxyglucose (18F-FDG) PET/CT Imaging Findings of Aggressive Systemic Mastocytosis: A Case Report

**DOI:** 10.7759/cureus.64879

**Published:** 2024-07-18

**Authors:** Mai Sato, Ryo Kurokawa, Naomasa Okimoto, Junji Tokushige, Masako Ikemura, Osamu Abe

**Affiliations:** 1 Radiology, The University of Tokyo, Tokyo, JPN; 2 Hematology and Oncology, The University of Tokyo, Tokyo, JPN; 3 Pathology, The University of Tokyo, Tokyo, JPN

**Keywords:** positron emission tomography, computed tomography, lymphadenopathy, anaphylactic shock, aggressive systemic mastocytosis

## Abstract

Aggressive systemic mastocytosis (ASM) is an advanced subtype of systemic mastocytosis characterized by organ involvement. In this article, we report a case with ASM in a 54-year-old woman with characteristic findings on computed tomography (CT) and fluorine-18-fluorodeoxyglucose positron emission tomography (^18^F-FDG PET)/CT. Contrast-enhanced CT on admission revealed hepatosplenomegaly, generalized osteosclerosis, colonic edema, edematous thickening of the wall in the ascending colon and edema in the surrounding regions of these organs and mesentery, ileus, subcutaneous edema, periportal collar sign, and multiple mesenteric lymphadenopathies. There was no ^18^F-FDG uptake in the lesions other than mild ^18^F-FDG uptake in the vertebrae, making the possibility of differential diagnoses such as metastasis, lymphoma, and extramedullary leukemia lower. Based on bone marrow biopsy results and clinical findings, the diagnosis of ASM was established. ASM can be a potentially fatal disease with a poor prognosis, and understanding its distinctive clinical course and imaging findings is crucial for early therapeutic intervention.

## Introduction

Systemic mastocytosis (SM) is a rare systemic disease characterized by abnormal proliferation and accumulation of mast cells in organs beyond the skin such as bone marrow, gastrointestinal tract, liver, and spleen [1,2]. The estimated incidence is 0.89 per 100,000 individuals [2]. SM is further classified into several subtypes based on bone marrow findings, B-findings (indicating mast cell proliferation) and C-findings (indicating organ involvement). Aggressive systemic mastocytosis (ASM) is an extremely rare and advanced subtype of SM with a median survival of 41 months, affecting only approximately 10% of patients with SM [3,4]. Based on the criteria outlined in the 2022 International Consensus Classification (ICC) and the fifth edition of the World Health Organization classification of tumors of hematopoietic and lymphoid tissues (WHO-HAEM5), ASM is defined by the presence of any of C-findings without ≥20% of immature atypical mast cells in bone marrow aspiration/biopsy (if met, diagnosis of mast cell leukemia should be made) or other associated myeloid neoplasm (AMN) (if present, diagnosis of SM with an AMN (SM-AMN) should be made) [4]. The establishment of the subtype is essential for appropriate risk stratification and management, and radiological examination such as computed tomography (CT) is a valuable non-invasive tool for comprehensively assessing the overall picture of a disease. However, due to the rarity of the subtype, imaging features of ASM have not been fully elucidated.

In this report, we present a case of ASM in a 54-year-old woman. Distinctive imaging findings at admission, combined with bone marrow biopsy and clinical presentation, led to the diagnosis of ASM.

## Case presentation

A 54-year-old woman presented with a history of soft stools for two years, progressing to watery stools five to six times daily over the past three months, prompting consultation with a previous doctor. She experienced a weight loss of approximately 10 kg over four months. Upper and lower gastrointestinal endoscopies revealed intestinal edema, aphthoid ulcers, and erythema, with a diffuse proliferation of cells containing c-KIT positive micro granules observed on biopsy, raising suspicion of SM, leading to referral to our hospital.

Physical examination at admission showed a temperature of 36.8℃, blood pressure of 150/86 mmHg, pulse rate of 102 beats/min, and SpO2 of 98% on room air. Rash and pitting edema of the lower legs were noted. Laboratory findings included mild anemia with hemoglobin of 10.7 g/dL (12.0-16.5 g/dL) and hypoalbuminemia with albumin of 2.9 g/dL (4.0-5.0 g/dL). Serum tryptase was elevated at 188 μg/L (1.2-5.7 μg/L) (Table 1).

**Table 1:** Laboratory findings on admission Mild anemia and hypoalbuminemia were noted. Hypokalemia was suspected to be due to diarrhea. Additionally, there was an elevation of tryptase, suggesting the proliferation of mast cells.

Parameters	Value	Normal range
White blood cell	6700 /μL	4000-9000 /μL
Neutrophil	72.6%	45.0-70.0%
Lymphocytes	22.6%	30.0-45.0%
Monocytes	4.3%	3.0-10.0%
Eosinophil	0.4%	2.0-10.0%
Basophil	0.1%	0.0-2.0%
Red blood cell	312x10,000 /μL	380x10,000-510x10,000 /μL
Hemoglobin	10.7 g/dL	12.0-16.5 g/dL
Platelets	18x10,000 /μL	15x10,000-35x10,000 /μL
Total protein	4.9 g/dL	6.7-8.3 g/dL
Albumin	2.9 g/dL	4.0-5.0 g/dL
C-reactive protein	0.18 mg/dL	<0.3 mg/dL
Total bilirubin	0.5 mg/dL	0.3-1.2 mg/dL
Aspartate aminotransferase	11 U/L	13-33 U/L
Alanine aminotransferase	17 U/L	6-27 U/L
Lactate dehydrogenase	136 U/L	119-229 U/L
Alkaline phosphatase	185 U/L	115-359 U/L
Gamma-glutamyl transpeptidase	77 U/L	10-47 U/L
Blood urea nitrogen	11.8 mg/dL	8-22 mg/dL
Creatinine	0.34 mg/dL	0.4-0.7 mg/dL
Sodium	145 mEq/L	138-146 mEq/L
Potassium	2.5 mEq/L	3.6-4.9 mEq/L
Chlorine	105 mEq/L	99-109 mEq/L
Calcium	6.9 mEq/L	8.7-10.3 mEq/L
Phosphorus	3.2 mEq/L	2.5-4.7 mEq/L
Tryptase	188 μg/L	1.2-5.7 μg/L
Soluble interleukin-2 receptor	1403 U/mL	122-496 U/mL
Interleukin-6	16.3 pg/mL	<7.0 pg/mL
Free-thyroxin4	1.01 ng/dL	1.1-1.8 ng/dL
Thyroid-stimulating hormone	4.37 μIU/mL	0.5-5.5 μIU/mL
Immunoglobulin G	667 mg/dL	870-1700 mg/dL
Immunoglobulin A	134 mg/dL	110-410 mg/dL
Immunoglobulin M	45 mg/dL	46-260 mg/dL
D-dimer	1.7 μg/dL	<1.0 μg/dL

**Figure 1:**
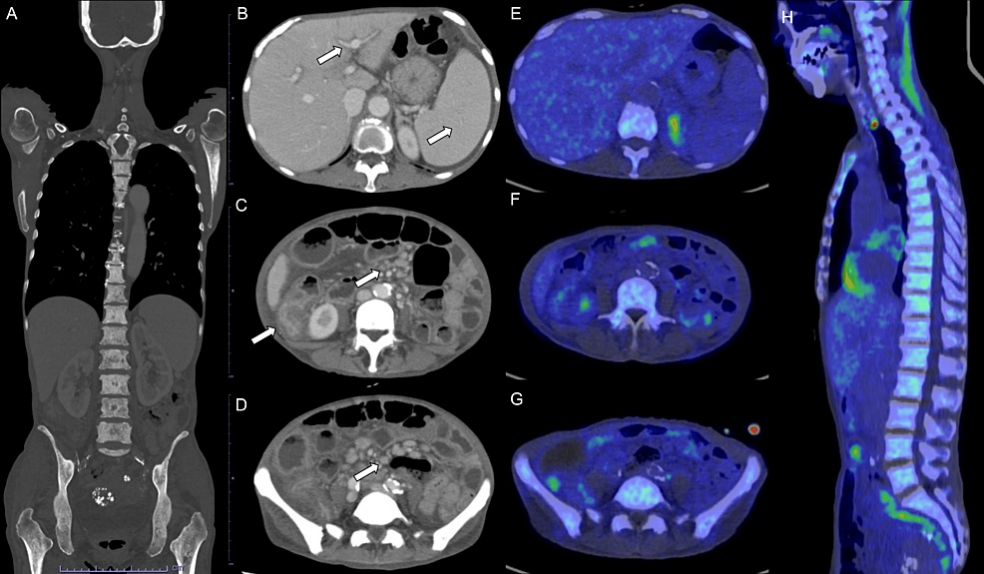
Computed tomography (CT) and fluorine-18-fluorodeoxyglucose positron emission tomography/computed tomography (18F-FDG PET/CT) imaging findings of aggressive systemic mastocytosis (A) Coronal image shows systemic osteosclerosis. Axial contrast-enhanced CT images show hepatosplenomegaly and periportal collar sign (B, arrows), edematous intestinal wall thickening with surrounding edema (C, arrow), multiple mesenteric lymphadenopathies with surrounding edema (D, arrow), and subcutaneous edema. Continuous dilation of the small intestine was observed without apparent obstruction, consistent with ileus. 18F-FDG PET/CT image slices corresponding to B (E), C (F), and D (G) show no abnormally increased 18F-FDG uptake in the lesions. Sagittal 18F-FDG PET/CT image reveals mild 18F-FDG uptakes in the vertebrae with a maximum standardized uptake value of 3.67 (H).

Contrast-enhanced CT at admission revealed systemic osteosclerosis (Figure 1A). Hepatosplenomegaly and periportal collar signs were evident (Figure 1B), along with edematous thickening of the wall in the ascending colon and edema in the surrounding regions of these organs and mesentery (Figure 1C). Multiple enlarged mesenteric lymph nodes were also observed (Figure 1D). Other than SM, polyneuropathy, organomegaly, endocrinopathy, M protein, and skin change (POEMS) syndrome; metastasis; malignant lymphoma; myelofibrosis; and other hematologic malignancy were considered as differential diagnoses.

To investigate malignancies, fluorine-18-fluorodeoxyglucose positron emission tomography/CT (^18^F-FDG PET/CT) was performed. ^18^F-FDG PET/CT did not show elevated ^18^F-FDG uptake in these enlarged lymph nodes, liver, spleen, intestine, or mesentery (Figures 1E-1G), supporting the diagnosis of SM. Mild ^18^F-FDG accumulation was noted in the vertebrae (Figure 1H), suggestive of the involvement of SM.

**Figure 2:**
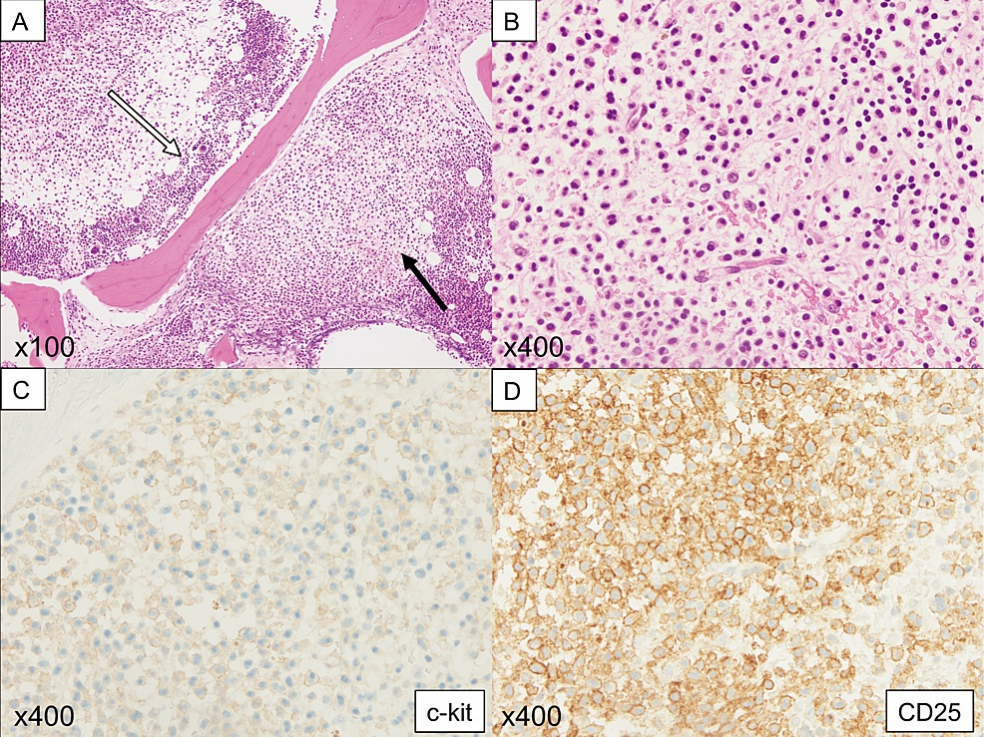
Histopathology of aggressive systemic mastocytosis (A, B) In hematoxylin and eosin staining, the proliferation of abnormal cells with minute granules (A, indicated by a black arrow and B) is observed alongside normal bone marrow tissue (A, indicated by a white arrow). The abnormal cells are positive for c-KIT (C) and CD25 (D).

Bone marrow biopsy performed after admission revealed abnormal cell proliferation with fine granules within the normal marrow tissue, with positivity for c-KIT and CD25, suggesting mast cell proliferation (Figure 2). The percentage of mast cells in the bone marrow was 4.2%, and the patient tested positive for the KITD816V mutation.

Based on these findings, the patient was diagnosed with ASM. Treatment with antihistamines to control anaphylactic shocks was initiated, but due to recurrent anaphylactic shocks, corticosteroids were introduced. Following corticosteroid therapy, there was no further occurrence of anaphylactic shock, and diarrhea symptoms improved. In the follow-up plain CT, the previously mentioned findings showed improvement. While consideration was given to invasive treatments such as allogeneic hematopoietic stem cell transplantation, the patient opted for pharmacotherapy alone, and as of seven months after the diagnosis, the patient is being monitored in outpatient care.

## Discussion

We report a case of ASM presenting with watery stool and weight loss of subacute onset. Contrast-enhanced CT revealed organomegaly, edematous intestinal wall, edema in the surrounding regions of involved organs, subcutaneous edema, and ileus. ^18^F-FDG PET did not show abnormally increased ^18^F-FDG uptake other than mild uptake in the vertebrae.

The latest consensus for the diagnosis of SM and further subtyping is based on the diagnostic criteria outlined by the WHO-HAEM5 and the 2022 ICC [5,6]. KITD816V mutation is detected in over 80% of SM cases in adults [1]. Multikinase inhibitors like imatinib have traditionally shown low activity against KITD816V mutation, prompting symptomatic treatment. However, newer options such as midostaurin and avapritinib have demonstrated efficacy against KIT mutations, providing new therapeutic choices [1]. In the present case, multifocal dense infiltrates of mast cells, positive for CD25 and exhibiting KIT gene mutation, were demonstrated in bone marrow and gastrointestinal biopsies. With a serum tryptase level of 188 ng/mL (>20 ng/mL), this case fulfilled one major and three minor criteria according to the WHO-HAEM5 criteria, leading to the diagnosis of SM [5]. Additionally, findings from biopsies indicated multifocal dense infiltrates of CD117-positive mast cells of bone marrow and other extracutaneous organs, fulfilling major criteria under the ICC classification [6]. SM is subdivided into several subtypes, including smoldering SM, indolent SM, ASM, SM-AMN, and mast cell leukemia [1]. In the present case, the absence of bone marrow dysplasia and mast cell involvement of less than 20% led to the diagnosis of ASM, supported by the presence of one C criterion (malabsorption with weight loss accompanied by hypoalbuminemia).

Bone lesions are most frequently observed in SM (up to 90%) [7]. Mast cells release various cytokines such as histamine, tryptase, tumor necrosis factor-alpha (TNF-α), and interleukin-6 (IL-6), which are implicated in affecting both osteoclasts and osteoblasts [8]. IL-6 has been associated with disease progression and bone stress [9], and in the present case, IL-6 levels were mildly elevated. Imaging findings often show a mixture of osteolysis and osteosclerosis, particularly affecting the axial skeleton, pelvis, and proximal ends of long bones [10,11]. These findings can sometimes be challenging to distinguish from metastatic bone tumors, lymphoma, or myelofibrosis [10].

Previous reports commonly indicate that ^18^F-FDG uptake is not observed in SM/ASM, but only observed in SM-AHN or mast cell sarcoma [11,12], although there are conflicting reports of strong ^18^F-FDG uptake in ASM cases [13]. Therefore, the absence of strong ^18^F-FDG uptake in the involved lesions can be a key finding in diagnosing SM/ASM, as observed in the present case.

Various gastrointestinal imaging findings have also been reported in SM/ASM, including hepatosplenomegaly, thickening, and dilation of the gastrointestinal tract with mucosal nodules or polypoid lesions [11,14]. Rare cases may show vascular complications such as Budd-Chiari syndrome or cavernous transformation of the portal vein [15]. Multiple enlarged lymph nodes in the retroperitoneum, mesentery, or around the portal vein have been observed, with reports indicating without or with increased ^18^F-FDG uptake in enlarged lymph nodes [13,14,16]. In the present case, hepatosplenomegaly, thickening of the ascending colon wall, and mesenteric lymphadenopathies were noted, without abnormal ^18^F-FDG uptake in enlarged lymph nodes.

Differential diagnoses of SM/ASM include POEMS syndrome; thrombocytopenia, anasarca, fever, reticulin fibrosis/renal dysfunction, and organomegaly (TAFRO) syndrome; malignant lymphoma; and myelofibrosis. While similar presentations such as osteosclerosis, organomegaly, and edema may overlap, the absence of multiple neuropathies, monoclonal plasma cell proliferation, or M protein can aid in the differentiation of SM/ASM from POEMS syndrome [17]. The absence of thrombocytopenia, increase of serum IL-6, serum vascular endothelial growth factor, serum alkaline phosphatase, or adrenal involvement may lower the possibility of TAFRO syndrome [18,19]. Additionally, the absence of strong ^18^F-FDG uptake in enlarged lymph nodes and the presence of physical signs related to increased vascular permeability may lower the possibility of malignant lymphoma and myelofibrosis.

## Conclusions

We present a case of ASM with characteristic CT and ^18^F-FDG PET/CT imaging features. Organomegaly, edematous intestinal wall, edema in the surrounding regions of involved organs, subcutaneous edema, and ileus with the absence or only mild uptake of ^18^F-FDG were suggestive of SM/ASM. Given the recent advent of novel therapeutics, a comprehensive understanding of these imaging manifestations is crucial for facilitating timely diagnosis, precise risk stratification of SM, and optimal patient management.
